# Genetic polymorphisms with erythrocyte traits in malaria endemic areas of Mali

**DOI:** 10.1371/journal.pone.0209966

**Published:** 2019-01-04

**Authors:** Karim Traore, Salimata Konate, Mahamadou A. Thera, Amadou Niangaly, Alhassane Ba, Alassane Niare, Charles Arama, Julie Di Cristofaro, Mounirou Baby, Stephane Picot, Jacques Chiaroni, Gilles Boetsch, Ogobara K. Doumbo

**Affiliations:** 1 Malaria Research and Training Center, DEAP/FMPOS, UMI3189, Université des Sciences, des Techniques et des Technologies de Bamako, Bamako, Mali; 2 Univ Lyon, Université Claude Bernard Lyon 1, Institut de Chimie et Biochimie Moléculaire et Supramoléculaire, UMR-5246 CNRS-INSA-CPE, Malaria Research Unit, Lyon, France; 3 Unité Mixte International UMI 3189 –Environnement—Santé—Sociétés, (CNRS/USTTB, CNRST/UGB/UCAD) Université Cheikh Anta Diop, Dakar, Sénégal; 4 Centre National de Transfusion Sanguine (CNTS), Bamako, Mali; 5 Aix-Marseille Université,CNRS, EFS, ADES UMR 7268, Marseille, France; Université Pierre et Marie Curie, FRANCE

## Abstract

African populations are characterized by high degree of genetic diversity. This high genetic diversity could result from the natural selection pressure. Several studies have described an association between some genetic diversities and difference of susceptibility to infectious diseases like malaria. It seems therefore important to consider genetic diversity impact when interpreting results of clinical trials in malaria endemic areas. This study aimed to determine the genetic polymorphism with erythrocyte traits in different populations of malaria endemic area in Mali. The cross-sectional surveys were carried out in different ethnic groups living in malaria endemic areas in Mali. Six milliliters of whole blood were collected in EDTA vials from each participant after informed consent has been obtained. The ABO, RH, Kell, MNSs, Kidd and Duffy systems phenotypes were assessed by the technique of gel filtration. A total of 231 subjects were included from six villages. The blood groups phenotypes O (40.7%) and A (31.2%) were more frequent with respective allele frequencies of 0.65 and 0.21. In the RH system the haplotypes R0 (0.55), r (0.20) and R1 (0.13) were the most frequent. Seven percent (7%) of Duffy positive and 4% of Glycophorin B deficiency (S-s-) were observed among participants. All participants were Kell negative. ABO and RH systems were polymorphic in these ethnic groups in Mali. Their implication in susceptibility to malaria should be taken into account in clinical trials interpretation, and for prevention of blood transfusion risks during anemia frequently caused by malaria in children.

## Introduction

The ABO and the other blood group systems remain of prime importance in transfusion medicine. These blood group antigens are mainly expressed on erythrocytes. They are the most immunogenic and the most common cause of life threatening from a blood transfusion in which an incompatible type of ABO blood is transfused. They are polymorphic and are associated to susceptibility/protection against certain infectious disease such as malaria.

Africa is a continent where higher population genetic diversity and the role of evolutionary processes that contributed to this diversity has been described [1,2]. It is also characterized by a lower degree of linkage disequilibrium [1,3], supporting the hypothesis of African origin of human. African genetic diversity is associated with significant demographic and cultural diversity, making the continent a relevant place to study factors contributing to the definition of gene pool of contemporary populations.

The Sahel-Saharan regions have a strong cultural diversity, with contrasting climatic areas in terms of rainfall, defining different environments and lifestyles. The populations living in these environments are constantly affected by numerous infectious diseases, leading to a strong pressure of natural selection [1,2]. This selection pressure results into a genetic diversity in populations, which may have diverse medical implications like susceptibility to disease.

Mali is a country with different eco-climatic areas and an important ethnic diversity. Some genetic diversities associated with differences in susceptibility to malaria have already been described in sympatric Malian populations [4,5,6,7,8]

Many clinical trials including therapeutic and vaccine studies are being conducted in such areas and blood transfusion is common due to severe malaria and sickle cell disease (reference). Studying the genetic structure of these populations (including erythrocyte systems) is crucial in interpreting the finding of clinical trials. In addition, the knowledge of genetic polymorphism map across the countries could have advantages in the prevention of risks related to blood transfusion in countries with limited resources where the technical platform cannot efficiently determine some erythrocyte phenotypes.

This study aimed to assess the genetic polymorphism with erythrocyte traits in populations living in different environmental and sociocultural conditions in Mali. We found that the ABO and RH systems were polymorphic in different ethnic groups in Mali. Therefore, their implication in susceptibility to malaria should be considered in clinical trials interpretation, and for prevention of blood transfusion risks during anemia frequently caused by malaria in children.

## Methods

### Study areas

**Fig 1:**
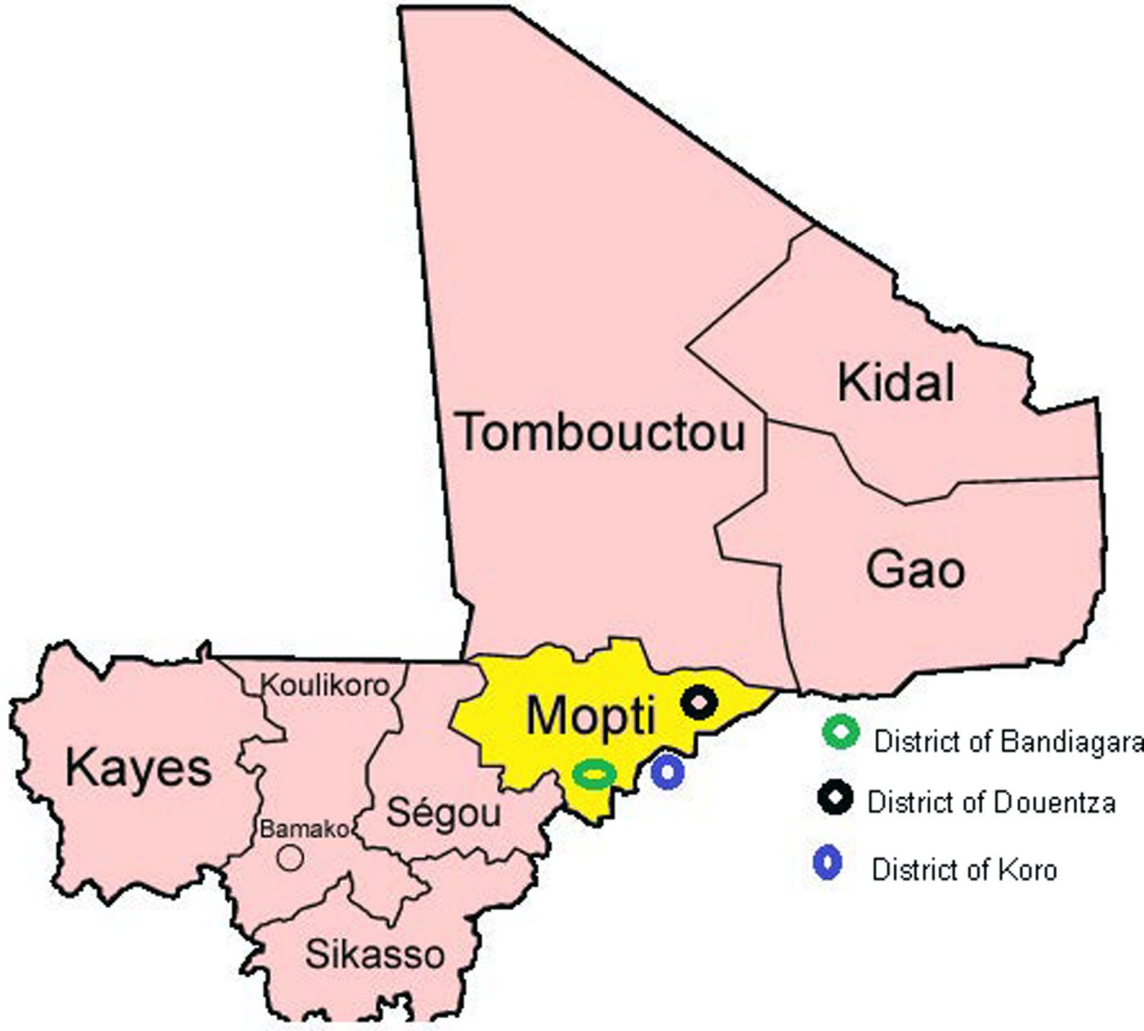
Map of Mali showing the different study sites.

The study was carried out in six villages with different eco-climatic and sociocultural conditions in the northeast of Mali. These six villages are located in three districts (Bandiagara, Koro and Douentza) in the region of Mopti (Fig 1).

Bandiagara: a city located at 700 km from the capital city in the northeast of Mali has 15,000 inhabitants. The population consists mostly of Dogon (64%) who live with Fulani and Tukulor (12%), and other ethnic groups. They practice ethnic exogamy and the caste endogamy. The languages are Niger-Congo-Atlantic, Niger-Congo-Mande and Afro-Asiatic-Berber.

Manteourou: a village with 1156 inhabitants (according to the census conducted in 2011) is divided into two districts (Dogon, Fulani) separated by approximately one kilometer.

Madougou: with 3128 inhabitants, is located at 12 kilometers from Manteourou and these two villages have the same configuration and characteristics (Madougou Dogon district and Fulani district).

Binedama: is a Fulani’s village with only 510 inhabitants (2011 census).

In these villages, the two ethnic groups (Dogon and Fulani) live in sympatry and practice ethnic endogamy. The language is Niger-Congo-Atlantic.

Petaka and N'Gono are located at around 150 km in the northeast from previous villages, in the district of Douentzan. Dogon constitutes the predominant ethnic group of the population. Family is mainly based on ethnic endogamy, patriarchal society, and the language is Niger-Congo-Atlantic.

The structure of these communities is patrilineal and patriarchal societies.

### Data collection

Data were collected in cross-sectional surveys from July to September 2011. A total of 231 participants from permanent resident populations were included in our study. Participants were included after they signed consent forms. As main inclusion criteria participant should be at least 3 years old, with no clinically chronic disease, no immune deficiency or history of blood transfusion in the last three months. A blood sample (6 ml in EDTA tube) was collected from each participant by venipuncture. Interviewing participants, we also collected the socio demographic data of each participant.

### Laboratory assay

Determination of blood group phenotypes: the manual technique of gel filtration (Ortho Bio Vue System, Ortho Clinical Diagnostics, France) was used for phenotyping of the blood groups systems according to the standardized operating procedure. Briefly, after opening filtration support, we added successively in each well one drop of saline solution, one drop of each reagent (Polyvalent human anti-globulins) and one drop of diluted blood with saline. A centrifugation was done for 5 minutes (Ortho BioVue System centrifuge), followed by 20 minutes incubation at 37°C, and then centrifugation for 5 minutes. For ABO system, diluted blood was directly added into the well of filtration support, which already contained the immunoglobulin anti-A and anti-B (+ anti-AB). The result was positive if there was agglutination or negative if there was no agglutination.

### Statistical methods

All data were entered and analyzed in SPSS software version 16. The phenotype frequencies were calculated for all study participants while allele frequencies were determined only in unrelated participants.

#### Determination of alleles frequencies

For ABO, Kidd, Duffy, MNSs and Kell systems, the Bernstein formula was used to estimate the frequencies of the alleles, with the assumption that the study population was in Hardy-Weinberg equilibrium.

#### Determination of the haplotypes frequencies in RH system

The algorithm EM (Expectation-Maximization) based on the maximum likelihood principle, and GENE [RATE] program were used to determine the genotypes of RH system.

## Ethical clearance

The study protocol was approved by the ethics committee ‘‘Comité d’étique de la faculté de médecine et d’ondontostomatologie” of Mali by the letter No. 2011-59-/FMPOS prior the study. Each participant signed two copies of informed consent form. The illiterate participants unable to sign used their fingerprint and were assisted by literate witness. All the study procedures were discussed in the participant’s desirable language in the presence of a witness. Parents or legal guardians signed the assent forms for the minors after having their consent. Each participant was given a signed copy of informed consent. To warrant the confidentiality, each participant was assigned a code, which has been used instead of the name on samples.

## Results

### Socio demographic characteristics of study participants

Overall 231 participants, composed in majority of Dogon (66,7%) and Fulani (29,4%) were included. Other ethnic groups (Tamashek and Bambara) constituted 3.9% of the sample. The sex ratio was 1.22 (males/females) and the median age was 38 years with respectively 3 and 88 years as minimal and maximal age. Binedama and Ngono villages had the lowest participant’s number with respectively 5.2 and 8.6% of participants (Table 1). Population structure is patriarchal and patrilineal.

**Table 1:** Baseline socio demographic characteristics of the study participants.

Village	Bandiagara	Madougou	Manterou	Petaka	N’Gono	Binedama	Total
**Dogon**	41	29	35	32	17	0	**154**
**Fulani**	13	25	17	0	1	12	**68**
**Others**	7	0	0	0	2	0	**9**
**Total**	61	54	52	32	20	12	**231**
**%**	26.4	23.4	22.5	13.9	8.6	5.2	**100**
**Median of age**		38 years					
**Maximal**		88 years					
**Minimal**		3 years					
***Sex ratio (M/F)***		1.22					

### Genetic polymorphisms of erythrocytes traits

**Fig 2:**
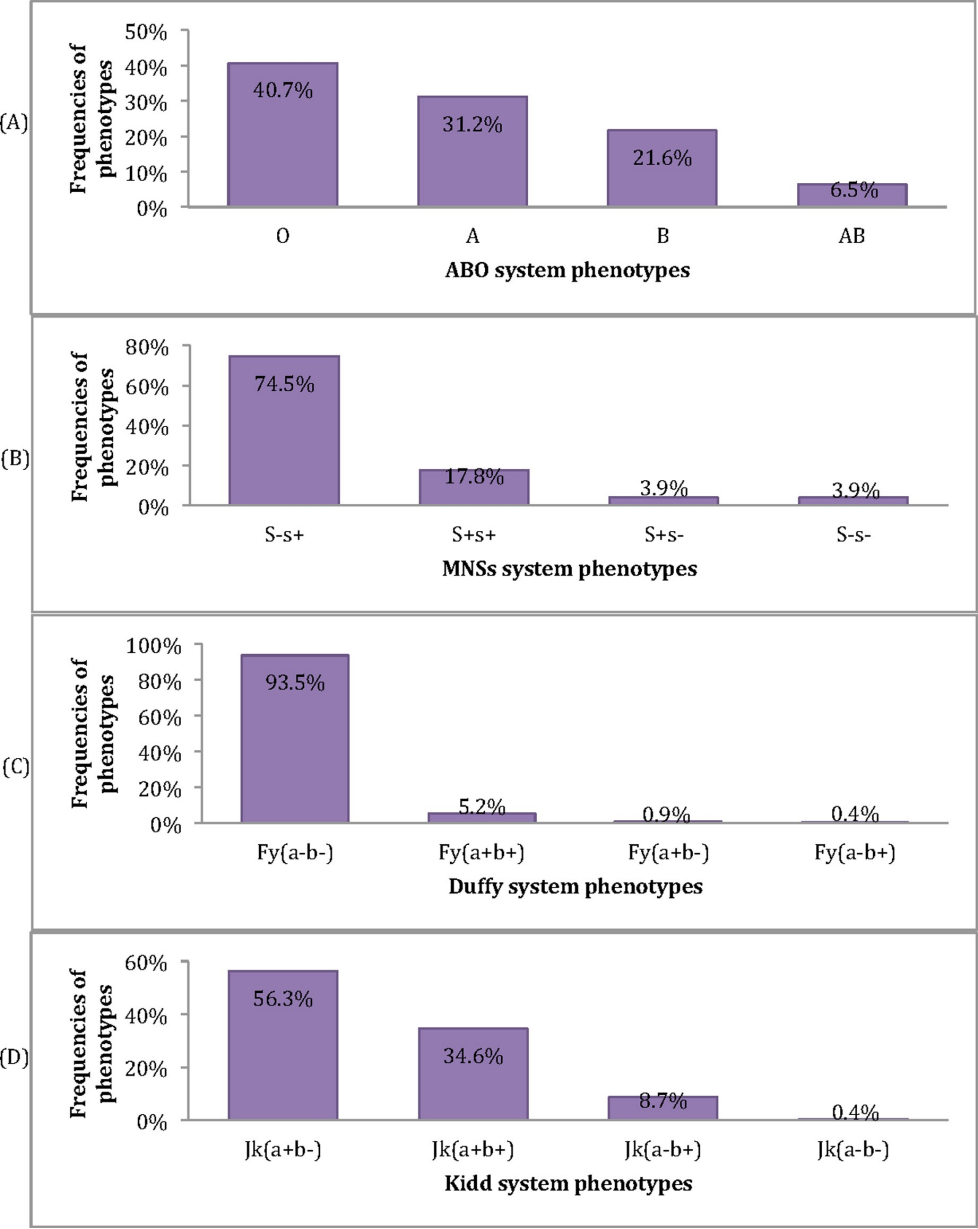
Distribution of phenotypes of ABO, MNSs, Duffy and Kidd systems in study population.

The phenotype O was the most frequent (40.7%), followed by A (31.2%), B (21.6%) and AB (6.5%) (Fig 2). The allele O (0.65) had the highest frequencies, followed by A (0.20) and B (0.14) for the ABO system (Table 2). In the RH system, the haplotype *R0* (0.55) was the most frequent, followed by *r* (0.20) and *R1* (0.13) (Table 3). Most of the study participants were Duffy negative with 93.5% of Fy(a-b-), and 6.5% of Duffy positive (Fig 2). The alleles frequencies were 0.99 for FY*O, 0.0065 for FY*A and FY*B (Table 2). In the Kidd system, the phenotypes frequencies were 56.3; 34.6; 8.7 and 0.4% respectively for Jk(a+b-), Jk(a+b+), Jk(a-b+) and Jk(a-b-) (Fig 2), with respective alleles frequencies of 0.75 and 0.25 for JK*A and JK*B (Table 2). Phenotypes frequencies in MNSs system were 74.5 for S-s+, 17.8 for S+s+ and 3.9% for S+s- and S-s- (Fig 2). All participants were Kell negative.

**Table 2:** Distribution of alleles frequencies of ABO, Duffy and Kidd blood systems in unrelated participants.

Blood system	Alleles	Frequencies
**ABO (N = 153)**	*A*	0.2037
	*B*	0.1482
	*O*	0.6517
**Kidd (N = 153)**	JK*A	0.7484
	JK*B	0.2516
**Duffy (N = 153)**	FY*O	0.99
	FY*A	0.0065
	FY*B	0.0065

**Table 3:** Distribution of haplotypes of RH system by ethnic group in unrelated participants.

Haplotypes	Fischer	*R0*	*R1*	*R2*	*r’*	*r”*	*Rz*	*r*
	CDE	*Dce*	*DCe*	*DcE*	*dCe*	*dcE*	*DCE*	*dce*
**Dogon (n = 109)**		0.621	0	0.092	0.072	0	0.019	0.195
**Fulani (n = 44)**		0.403	0.362	0	0	0.019	0.025	0.189
**All (n = 153)**		0.552	0.134	0.069	0.032	0.016	0.011	0.20

## Discussion

The study involved populations from six villages with different eco-climatic conditions and lifestyles. The lower numbers of participants from Binedama and N’gono are due to small total population size of these villages. Most of the study sites were Dogon and/or Fulani villages, leading to a high representation of these two ethnic groups in our sample. Except children under 3 years old, all the age groups (3–88 years) were represented in our sample, allowing the determination of erythrocytes polymorphisms distribution at all ages. Children less than 3 years old were excluded from the study to avoid confounder factors related to blood group systems immaturity.

### ABO system phenotypes and alleles distribution

High frequencies of blood group O phenotypes in Sub-Saharan Africa are already reported in literature [9,10]. In Mali, a recent survey in the national blood transfusion center reported 42% of O phenotypes [8]. Another study which included five ethnic groups in malaria endemic area in Ivory Cost in 2009 reported 50–60% of O phenotype [6]. The predominance of this phenotype in Sub-Saharan Africa has been suggested to be related to the natural selection pressure by some endemic diseases like malaria [4,7,11,12,13]. The rosetting mechanism has been described as one of the factors that contributed to the positive selection of O allele carriers by conferring to them relative resistance to malaria [4]. However, demographic phenomena including populations’ migration, genetics drift and the founder effect could also have considerable role in the distribution of erythrocyte phenotypes. The Mitochondrial DNA and Y-chromosome analysis could contribute to determine the role of these demographic phenomena in the actual distribution of erythrocytes polymorphism [10].

The highest frequencies of O phenotype were observed in Fulani ethnic group with 48.6% (Table 4). Reduced susceptibility to malaria has been described in Fulani populations, compared to their neighbors’ sympatric Dogon and Mossi populations [5,6,8]. The contribution of antigen presenting cells such as dendritic cells and Toll-Like Receptors activation pathways, or polymorphism of gene coding for some cytokines have been largely investigated in this difference of susceptibility to malaria [5,6,8]. But the real causes are still non-elucidated. The high frequencies of blood phenotype O in Fulani ethnic group could play a role in this difference of susceptibility.

**Table 4:** Distribution of phenotypes of blood systems MNSs, Kidd, Dufy and ABO by ethnic groups.

Blood system	Phenotypes	Frequencies by ethnic groups			P
		Fulani	Dogon	Others	
MNS (N = 231)	S^-^s^-^	7.4% (5)	2.6% (4)	0	
	S^+^s^-^	0	5.8% (9)	0	
	S^+^s^+^	19.1%(13)	18.2% (28)	0	
	S^-^s^+^	73.5% (50)	73.4% (113)	100%(9)	
Kidd (N = 231)	JK(a^-^b^-^)	0	0.6% (1)	0	
	JK(a^+^b^-^)	47.1% (32)	61.7% (95)	33.3% (3)	
	JK(a^+^b^+^)	36.8% (25)	31.8% (49)	66.7% (6)	
	JK(a^-^b^+^)	16.2% (11)	5.8% (9)	0	
Duffy (N = 231)	Fy(a^-^b^-^)	98.5% (67)	92.9% (143)	40% (4)	
	Fy(a^+^b^-^)	0	0	40% (4)	
	Fy(a^+^b^+^)	1.5% (1)	7.1% (11)	0	
	Fy(a^-^b^+^)	0	0	20% (2)	
ABO (N = 231)	A	27.9% (19)	32.5% (50)	33.3% (3)	<10^−3^
	B	17.6% (12)	24% (37)	11.1% (1)	
	AB	5.9% (4)	6.5% (10)	11.1% (1)	
	O	48.6% (33)	37% (57)	44.4% (4)	
Total		68	154	9	

### The RH haplotypes distribution

The haplotype *R0* had the higher frequencies in our study population. Previous studies have described the high frequencies of *R0* in Sub-Saharan populations [14–16]. This may be consistent with the fact that Rh haplotype is the ancestral one and as it is seen with other haploidic systems (mDNA and Y chromosome), the most ancient is found in Africa considered as cradle of modern human [15,16]. The prevalence of HbS allele which is another genetic polymorphism conferring resistance to malaria [17,18], may determine the distribution of haplotype *R0*. Carriers of this allele HbS have enhanced expression of the antigens D, c et e, leading to the high frequencies of haplotype *R0* in high sickle cell prevalence area [14,15]. Dogon people, who are known to have a much lower frequency of the HbS allele, have in contrast a high frequency of the HbC allele [17]. All these genetic markers polymorphisms are the result of natural selection and co-evolution of human and malaria parasite [18].

Higher frequency of haplotype *R1* was described in this study, compared to previous data published [16]. This haplotype was present exclusively in Fulani population Table 3). The Fulani population practice ethnic endogamy, which establishes a genetic distance between them and other ethnic groups. These data could also support the back migration «back to Africa» hypothesis reported in literature [19–22].

### Duffy system phenotypes and alleles distribution

As already described in black populations [7,9,16,18], we found higher frequencies (93.5%) of Fy(a-b-) phenotype and FY*O allele (0.99) in our sample. However, 6.5% of our participants were Duffy positive. Menard *et al*. described 8.8% of asymptomatic *Plasmodium vivax infection* in Duffy negative people of Madagascar where 28% of the population are Duffy positive [23]. Recently in Mali, during a cohort study including 400 children, 2.5% of *P*. *vivax* infection have been described [24]. The low frequencies of Duffy positive in African populations could be a factor that maintains a silent transmission of *P*. *vivax*. *P*. *vivax* may also develop alternative mechanism to bypass the obstacle due to Duffy binding protein deficit to infect erythrocyte. Investigating this new mechanism used by *Plasmodium vivax* to infect Duffy blood negative erythrocytes is crucial for malaria control and elimination. Malaria control programs should incorporate more sensitive tools for the diagnosis and surveillance of *P*. *vivax* in malaria endemic areas.

### MNSs system phenotypes distribution

The phenotype S-s+ (74%) was the most frequent in our study participants. Higher frequency of Glycophorin B deficiency (S-s-) was observed in Fulani (7.4%), compared to their neighbor Dogon (2.6%) (Table 4). The Glycophorin B deficiency is described as natural resistance factor to malaria infection invasion [23]. Recent multicenter studies of Malaria Gen consortium have also documented the role of glycophorines deficiency as naturel resistance factor to malaria [25]. The presence of this phenotype in malaria endemic areas could be a result of natural selection pressure from malaria. However, these phenotypes need to be confirmed by molecular assay.

## Conclusion

Polymorphisms among the different populations were mainly observed in the ABO and RH systems. The ethnicity and the environment of residence were the factors involved in polymorphism distribution; highlighting the role of environmental natural selection in the genetic diversity. The study of erythrocytes polymorphisms is a significant contribution in understanding the differences in susceptibility to malaria. We have described in Fulani ethnic group higher frequencies of O blood phenotype and glycophorines B deficiency known as genetic factors conferring natural protection against malaria. The natural selection pressure can involve several genetic markers (including erythrocytes systems thought to be sensitive to natural selection). The natural resistance to disease could be the sum of polymorphisms of several genetic markers. Since some polymorphisms in erythrocyte systems have been described to be associated with susceptibility or resistance to diseases, their distribution and their impact should be considered in epidemiological studies of malaria. For an accurate interpretation of clinical trials data for efficacy of drugs or vaccine in malaria endemic areas, any ethnic group difference in the distribution of erythrocytes phenotypes should be considered. The knowledge of erythrocytes polymorphism countrywide could also contribute to reduce risks related to blood transfusion.

## Supporting information

S1 File Database. (SAV)
